# “I’m Afraid to Put Any More of It Into My Body”: COVID-19 Vaccination and Booster Barriers and Facilitators Among People with HIV in South Carolina

**DOI:** 10.1007/s10461-025-04642-w

**Published:** 2025-03-03

**Authors:** Camryn Garrett, Arielle N’Diaye, Shan Qiao, Xiaoming Li

**Affiliations:** Smartstate Center for Healthcare Quality, Department of Health Promotion, Education, and Behavior, Arnold School of Public Health, University of South Carolina, Columbia, USA

**Keywords:** COVID-19, Vaccination, Hesitancy, People with HIV, Qualitative study

## Abstract

As people with HIV (PWH) have an immunocompromised status and face potential complications from a COVID-19 infection, there are alternate, more expansive, vaccination schedules recommended for PWH. As the pandemic evolves and prevention fatigue rises, the vaccination sentiments and hesitancy of PWH require attention amid continued recommendations for boosters. Situated within South Carolina, this study aims to illustrate PWH’s vaccination sentiments, as well as barriers and facilitators to vaccination. Semi-structured interviews were conducted online between March and August of 2023, among 24 PWH who were snowball and purposively sampled at a local AIDS Service Organization. An abductive approach was employed. All interviews were recorded, transcribed, and coded using an inductive, thematic analysis approach to identify and analyze emergent themes, which were then deductively categorized into the socioecological model. At the individual level, the need to protect oneself and others, prioritization of vaccination due to HIV status, and a positive personal history of vaccination facilitated uptake while a negative personal history acted as a barrier. Within the interpersonal and institutional level, family and friends as well as healthcare providers were found to serve as both positive and negative vaccine messengers. At the structural level, vaccine requirements and mandates (e.g., employer, travel) facilitated uptake while misinformation, misunderstanding, and skepticism (e.g., pace and process of vaccine development) acted as barriers to uptake. Tailored vaccination education and enhanced trust between providers and PWH may improve vaccination sentiments and mitigate hesitancy, as additional doses continue to be recommended.

## Introduction

In response to the COVID-19 pandemic, vaccines were rapidly developed to mitigate the risks of morbidity and mortality associated with infection [1]. In the winter of 2020, vaccine distribution began with priority given to frontline healthcare workers as well as to immunocompromised and elderly populations [2]. Among people with HIV (PWH), those who were not virally suppressed or not engaged in care were prioritized for vaccination initiation and booster uptake to avoid the potential morbidity and mortality associated with COVID-19 infection. The prioritization of PWH in vaccination was prompted by historic and emergent disparities. During the COVID-19 pandemic, PWH were found to disproportionately experience adverse pandemic-related outcomes (e.g., increased severity of infection, hospitalization, death after hospitalization, HIV progression), in comparison to people without HIV [3–5]. Therefore, there is a clear and present need to prioritize pandemic prevention (e.g., vaccination) in efforts to reduce the morbidity and mortality experienced by PWH.

Although vaccination remains the most promising method for pandemic prevention, misinformation and vaccination hesitancy have been found to result in vaccination delays or avoidance [6, 7]. A systematic review of the determinants of vaccination in the United States found that acceptance ranged between 53.6% and 84.4%, with variable acceptability found among certain demographic groups (e.g., decreased acceptability once vaccines were available among young, Black, Hispanic, low income, and republican populations) [8]. The presence of underlying medical conditions have been found to be positively associated with an intention to vaccinate [8]. As related to HIV, by the fall of 2021, in New York, about 63.5% of PWH had completed the initial COVID-19 vaccination series, while 32.2% remained unvaccinated [9]. Lower vaccination coverage was found among racial and ethnic minority PWH, those who were not virally suppressed, and those not engaged in HIV care [9]. Further, a global systematic review and meta-analysis of COVID-19 vaccination hesitancy among PWH found a hesitancy prevalence of 34% with variation due to gender, race/ethnicity, region, influenza vaccination history, and HIV viral suppression status [10]. As patterns in vaccination uptake and hesitancy emerge, there persists a need to understand the perspectives and experiences of PWH who face vaccination disparities.

Factors across the socioecological model (i.e., individual, interpersonal, institutional, and structural levels) influence vaccination decision-making, hesitancy, and uptake. The socioecological model situates individuals within their broader socio-environmental contexts to demonstrate the relationships between an individual and the systems under which they live [11]. A scoping review of the international COVID-19 vaccination literature identified factors associated with vaccine acceptance or hesitancy, at the interpersonal and institutional levels, as including health communication message frames, opinions and uptake of family and friends, physician recommendations, anti-vaccination messages, and disinformation in media (i.e., traditional media, social media) [12]. A meta-analysis, of studies of vaccination among PWH, found a higher odds of vaccine acceptance among those who perceived themselves as at-risk of complications from an infection and reported believing in the vaccine’s effectiveness [13]. Among PWH in the United States, vaccine uptake prevalence was found to be 71.6%, but there persist disparities in uptake among subpopulations of PWH (i.e., women with HIV, Black PWH, Latino PWH, PWH with primary education or below, unemployed PWH, PWH with unsuppressed viral loads) [13, 14]. Disparities in vaccine acceptance and uptake are perpetuated across the socioecological model [13, 14]. Vaccination access and decision-making is a multifaceted process that requires an in-depth understanding of the impact of factors within and beyond individual control.

As additional doses and boosters of the COVID-19 vaccination are recommended for PWH, further investigation into vaccination sentiments, among PWH living in regions characterized by vulnerability and vaccination hesitancy (e.g., Deep South), is warranted [2, 15–17]. PWH living in the Deep South face unique vulnerabilities due to their geographic location (e.g., distance to healthcare facilities), differential access to healthcare (e.g., limited access to telehealth, insurance status), and racial inequities (e.g., racism, health disparities), that are exacerbated in times of public health crisis (e.g., COVID-19) [18]. An in-depth understanding of the barriers and facilitators to vaccination is needed for the normalization of seasonal vaccines (e.g., influenza, potentially COVID-19) and in future infectious public health emergencies (e.g., monkeypox, avian flu).

The present study, therefore, responds to calls in the literature for work focusing on the perspectives of PWH, particularly those from various racial and ethnic groups and educational backgrounds [19, 20]. Topically, this work is in response to calls in the extant literature for research that further explores how hesitancy impacts booster uptake, the role of skepticism in vaccine decision-making, and the perspectives of PWH, more broadly, to inform tailored public health efforts [10, 14, 19, 20]. As vaccination (i.e., initial series and boosters) uptake remains a primary tool for pandemic and infectious disease prevention (e.g., COVID-19, influenza, monkeypox), the present work serves to inform future policy and intervention implementation within the Deep South. Addressing these literature gaps, the present work is guided by the research question: *What are the hesitancies and perceived barriers and facilitators of COVID-19 vaccination among PWH in South Carolina?*

## Methods

### Study Design

Participants were purposively and snowball sampled. Participants were recruited by flyers and provider referral, at a participating local AIDS Service Organization (ASO), and by participant referral. Those eligible to participate in the study were those aged 18 years or older, living in South Carolina, and who had lived with HIV for 2 or more years at the point of recruitment. This recruitment strategy yielded 24 participants. Study activities were conducted in alignment with the Declaration of Helsinki and the Belmont Report. The study protocol was reviewed and approved by the Institutional Review Board at the University of South Carolina (Pro00114275). All methods and findings are presented in accordance with the Consolidated Criteria for Reporting Qualitative Research (COREQ) [21].

### Data Collection

All interviews were conducted by an author trained in qualitative research methods, who holds a Master of Public Health, and was a graduate research assistant at the time of the study (CG). There were no established relationships with participants prior to the commencement of the study. There was limited participant knowledge of the interviewer beyond the primary aims of seeking to understand the lived experiences and perspectives of PWH as related to COVID-19 and vaccination. All interviews were conducted one-on-one over the teleconferencing software Zoom and followed a semi-structured interview guide [22]. Prior to participation in the interview, all participants provided their written informed consent and verbally consented to being recorded. The informed consent procedure described the study activities, protections and risks to participant privacy, and the fully voluntary nature of all activities, where participants were able to withdraw at any time, for any reason. Interviews were conducted between May 2022 and August 2023, with rolling enrollment, and were between 30 and 60 min in duration. Each participant was compensated up to $120 USD for their time and efforts participating in the interview alongside additional study activities (i.e., ecological momentary assessment). No further interviews were conducted once saturation was reached [23]. All interviews were recorded and uploaded to the AI transcription software Otter.ai (2024) [24]. Once transcribed, a member of the research team (CG) cleaned the transcripts to ensure that all data were deidentified and transcribed verbatim (i.e., compared each text file to its audio recording).

### Data Analysis

The analysis was conducted following an abductive approach, where the data were first inductively coded and then emergent findings and latent level extractions were situated, deductively, within the socioecological model [25, 26]. The data were initially analyzed following an inductive, emergent thematic analysis approach. The thematic analysis followed the 6 phases of analysis, set forth by Braun and Clarke, to (1) familiarize yourself with your data, (2) generate initial codes, (3) search for themes, (4) review themes, (5) define and name themes, and (6) produce the report [27]. The data were initially coded by the same author who conducted the interviews (CG) and were then reviewed by two additional authors upon completion of each phase of coding (AN, SQ). Upon completion of the inductive coding, the research team found that the emergent themes mirrored the various levels of the socioecological model, which then led to their deductive categorization, as appropriate. All authors reviewed and discussed the analysis matrix, which was comprised of sub-categories, categories, subthemes, and themes (Appendix). All authors kept a reflexive journal, and an audit trail was maintained throughout the data analysis process. All data were managed and analyzed using Excel [28]. Exemplary quotations were extracted from the interview transcripts to illustrate emergent themes.

## Results

The present findings are based on interviews with 24 PWH, in South Carolina, during the COVID-19 pandemic. The average age of the participants was 48.92 (SD = 10.68) and ranged from 30 to 71 years of age. Most participants identified as Black (75%), were employed full-time (45.83%) or part-time (16.67%), had been living with HIV for an average of 18.92 years (SD = 7.84), had not ever had COVID-19 (66.67%), and had received an average of 3 (SD = 1) COVID-19 vaccinations (Table 1).

**Table 1:** Demographic characteristics of the sample

Variables	Mean (SD) or *n* (%)
**Age**	48.92 (10.68)
**Gender**	
Women	14 (58.33%)
Men	10 (41.67%)
**Race/ Ethnicity**	
Black	18 (75%)
Hispanic	1 (4.17%)
Black & Hispanic	1 (4.17%)
White	4 (16.67%)
**Education**	
High School or Equivalent	6 (25%)
Associates	4 (16.67%)
Some College	9 (37.5%)
Bachelors	2 (8.33%)
Some post Bachelors	1 (4.17%)
Masters	2 (8.33%)
**Employment**	
Full-time	11 (45.83%)
Part-time	4 (16.67%)
Unemployed	2 (8.33%)
Retired	1 (4.17%)
Receiving Disability	6 (25%)
**Years Living with HIV**	18.92 (7.84)
**Ever have COVID-19**	
Yes	8 (33.33%)
No	16 (66.67%)
**COVID-19 Vaccinations**	3 (1)

Emergent themes were categorized using the socioecological model (i.e., individual, interpersonal, institutional, structural). Overall, the findings demonstrate that the vaccination hesitancy and booster uptake of PWH is influenced by the perceptions and uptake of people within their social networks, healthcare providers, the availability of resources, and structural perpetuations of misinformation and misunderstanding. At the individual level, facilitators of vaccination uptake were the need to protect oneself and others, prioritization of vaccination due to HIV status, and a positive personal history of vaccination experiences, while negative vaccination experiences were a barrier. Within the interpersonal level, family and friends acted as both positive and negative vaccine messengers, both facilitating and deterring vaccination, respectively. Similarly, within the institutional level, healthcare providers also acted as both positive and negative vaccine messengers. At the structural level, facilitators included ease of vaccine access as well as vaccination requirements and mandates set forth by organizations and institutions, while barriers included difficulty accessing vaccines, misinformation and misunderstanding, as well as hesitancy induced by the pace and process of vaccine development (Appendix).

### Individual Level

Vaccination decision-making among PWH was found to be facilitated and obstructed by a variety of factors at the individual level. Vaccination was facilitated by the need to protect oneself and others, HIV status, and a personal history of positive experiences with vaccines. Alternatively, a personal history of negative experiences with vaccines impeded participants’ willingness to vaccinate.

### Facilitator: Need to Protect Oneself and Others

Despite their hesitancy, participants discussed the motivation behind their choice to get vaccinated as due to their beliefs in vaccines and their ability to protect the recipient against the morbidity and mortality potentially associated with a COVID-19 infection. One participant shared this perception when they said, *“I did at first. But then I’m like*,* it’s my life and I am high risk. I need to know what I need to do to get myself protected.”* (Participant 2). Relatedly, a participant described the need to vaccinate themselves and their household when they said, *“I can’t play with my life. I really can’t. So*,* I was like*,* I gotta go get it. So everybody in the house has been vaccinated.”* (Participant 11). A participant described how the potential benefits of the vaccine outweighed fear and skepticism over the efficacy of the vaccine when they shared, *“But I said*,* you know*,* I’m gonna go ahead and do it. Because even if it does nothing*,* yeah*,* it’s*,* it’s a step towards trying to help protect*,* not only myself*,* but my mom and my sister and my niece and my*,* my close friends.”* (Participant 19). Similarly, a participant also stated, *“…people were getting sick and dying right after the vaccination. Well*,* guess what? You’d rather be trying to get the vaccination and be protected than to not get it and still have a risk.”* (Participant 2). Further, one participant described the importance that others in their community get vaccinated as a motivation to vaccinate themself. This was found when they shared, *“So I got the first one. Because I needed my communities to get it. I’m like*,* okay*,* I want them to get it. I’ve got to take it.”* (Participant 22).

### Facilitator: Vaccination Prioritization Due to HIV Status

Participants shared that their HIV status was a facilitating factor in their uptake of the COVID-19 vaccine and boosters. One participant shared the avoidance of HIV complications as a motivator to take the vaccine because, as they said, *“I don’t want nothing to trigger my HIV.”* (Participant 15). Other participants described initially experiencing vaccination hesitancy but ultimately decided to get vaccinated. For instance, one participant described, *“And then I was like*,* no*,* that’s not*,* not for me. And dealing with the HIV*,* and I’m like*,* okay*,* is it going to make me more or is it gonna make me less of like*,* I was just back and forth*,* back and forth.”* (Participant 11). Another participant noted, *“So I was really kind of anxious to get it though*,* because I wanted to make sure that I was protected just for HIV.”* (Participant 1). Additionally, one participant described the influence of regularly taking a long-acting antiretroviral injectable, as part of their HIV treatment, on their COVID-19 vaccination uptake when they described, *“I said I’m gonna go. Might as well get it because I’d already been taking these Cabenuva shots and other stuff. So*,* I say*,* you know*,* one more thing. I had the monkeypox vaccine too. So I’m going to be a walking*,* walking petri dish after a while*,* I guess.”* (Participant 13).

### Barrier: Personal History of Negative Vaccination Experiences and Side Effects

A few participants shared negative experiences of side effects after receiving a COVID vaccination or booster. One participant described the impact of the side effects when they shared, *“But it had*,* it*,* it made me feel bad…The second booster made my bones hurt. I was in the bed for about two days after I took that second booster.”* (Participant 9). Another participant, who had not yet received a booster, described their experience with the initial doses of the vaccine when they said,

“I wasn’t [hesitant] with the first one…20 minutes later… I’m like, oh, I don’t feel good. When I told my mom, was like, ‘Mom, I got a shot, but I’m going to lay down. I don’t feel good.’ She’s like, ‘You shouldn’t have taken it, go lay down.’ So, when the other one was coming up with like, I don’t know. I don’t know, want to go through that… Yeah, my reaction wasn’t that nice. So, when I had to take the second one, I was very hesitant, but then my reaction to that one wasn’t, wasn’t, it’s like, I didn’t even take a shot.” (Participant 23).

### Interpersonal Level

#### Facilitator: Family and Friends as Positive Vaccine Messengers

Participants detailed the facilitating impact of their family and friends’ supportive opinions and positive experiences with the vaccine on their decision-making. One participant shared the importance of the choices of those within her social network when she said, *“They got it so*,* you know*,* so that made it easier. And then on top of it*,* so many women you know*,* in my network*,* and they got it.”* (Participant 9). Another participant described the influence of their family’s decision to vaccinate, and which vaccine to choose, on their uptake when they shared, *“So I went with their decision. They had done already got it. Like they was*,* like*,* the very first ones when it hit*,* they got it. Yeah*,* so I was like*,* ‘Yeah*,* which one y’all went with? Pfizer? Yeah*,* I’m on my way right now.’”* (Participant 6). Participants noted the motivating impact of the positive vaccination experiences of others around them when they detailed, *“So I said ‘Okay*,* well you didn’t have any side effects and you’re older.’ So*,* telling me to go ahead and get mine. And so*,* I feel*,* I felt a little better talking to the folks who had had it already.”* (Participant 13). Additionally, the choice of one participant’s romantic partner to vaccinate motivated their vaccination decision-making: *“He just was like*,* ‘Yeah*,* I’m getting one whether you do or not*,* I’m getting one.’ You know*,* he was trying to*,* you know*,* press upon me to get one but he was doing it for himself.”* (Participant 14).

Further, health concerns expressed by participants’ family and friends facilitated vaccination uptake. For instance, one participant shared:

“My mom [was] yelling at me back and forth. She’s like, ‘You’re already sick. And it can help. So, you might as well just go ahead and get it done. You got to go to the doctors anyway.’ I’m like, ‘You know what, fine.’ I got tired of hearing her voice too. And I’m like, ‘You know what, fine. I want to get it done.’” (Participant 11).

Another participant described receiving encouragement to vaccinate from her husband who did not intend to vaccinate himself. She shared:

“Um, he said, what he says is, ‘I’ve never had the flu, so I’ve never taken a flu shot.’ So that’s how he looks at it. But he’s more so, ‘Oh, you’re gonna get it done. You’re gonna do this, you’re gonna do that. Because we can’t take chances with you because we don’t know what you can fight and what you can’t fight.’ So, I was hesitant at first about getting it done. But once I thought about it, go ahead. Oh, well.” (Participant 21).

#### Barrier: Family and Friends as Negative Vaccine Messengers

In addition to family and friends serving as positive vaccine messengers, participants also shared instances of them acting as negative messengers. The negative experiences of others with vaccination, and related side effects, acted as a barrier to vaccination. One participant demonstrated this when they shared their hesitation as resulting from, *“Just hearing the side effects and then what my [friend] went through with [it]*,* she broke out in a very bad rash…Like*,* very bad. And then I was like*,* no*,* that’s not*,* not for me.”* (Participant 11). Another participant described their hesitation as due to the conflicting perspectives of others: *“I was [hesitant] because some people gave the vaccine negative reviews and then some people [said] it ain’t that bad.”* (Participant 5). One participant described the role of her husband’s opinions on her ability to get vaccinated when she shared, *“We’re old school. He. Yes. I mean*,* I didn’t get [it] until he was like*,* ‘Okay. I’m cool with it.’ He thought the government was like injecting stuff in us. You know that whole talk. He’s a caveman. But um*,* yeah*,* we*,* we did it. I said*,* like*,* ‘I have to get it because like*,* I’m in*,* I already have a compromised system.’”* (Participant 20).

Further, participants described the negative influence of vaccination stigma, within their social networks, on their decision to vaccinate and to share their vaccination status. For instance, one participant described, *“And I was real funny about taking it just because of the stigma and stuff I was hearing from my family. It wasn’t nothing that I was seeing. It was just that that was being fed to me.”* (Participant 16). Participants further described vaccination hesitation within their social networks, such as when one participant shared, *“Some of my friends were just like*,* ‘I’m not*,* I’m not doing it. The vaccine don’t work.’ Of course. Like the flu shot. They say the flu shot is giving them the flu so the COVID vaccine is going to give them COVID.”* (Participant 8). As a result of vaccination stigma, participants also concealed their vaccination status from their family and friends. One participant shared, *“Well*,* there’s certain people I never told. Like*,* I never told my dad I got it.”* (Participant 22). An additonal participant described concealing their vaccination status when they shared:

“And I didn’t even tell them I was vaccinated until, you know, I got COVID. I said, ‘Well, I was vaccinated.’ But I was afraid to tell them I was vaccinated because of how they felt about it. I would not have dare told them that, ‘Hey, I’m going to get vaccinated,’ because they probably would have tried to talk me out of it or probably wouldn’t, wouldn’t talk to me. It’s, like, because I went and got vaccinated.” (Participant 7).

### Institutional Level

#### Facilitator: Healthcare Providers as Positive Vaccine Messengers

Participants described the influential role of healthcare providers, particularly infectious disease providers, in facilitating vaccination uptake. One participant detailed the impact of their trust in their healthcare provider as mitigating their vaccination hesitancy when they shared:

“No, because everybody else didn’t want to do it, but I know, oh, the health issues that I have. My doctor was like, ‘You need to get it’…Yeah, ’cause I always listen to my doctors. Even though I know I drive them crazy. I always listen to my doctors…They treat me like, they treat me like a patient, but they also treat me like a family member too.” (Participant 12).

Further, a participant described the normalization of vaccinations, due to their integration into their HIV care, when they shared, *“The very first ID [infectious disease] doctor that I had*,* he was such a sweetheart. You know*,* he introduced me to the new norm of the vaccine and told me how important that these things were*,* that I need*,* because of my condition. So*,* they say to us*,* ‘When they say you need a vaccine*,* don’t you fight them.’”* (Participant 4). Another participant described the positive influence of discussions with their healthcare providers on their decision to vaccinate when they described:

“It was, well, he was telling me to get it. I basically was telling him no at first. Like I said, it took about two or three weeks. Um, we talked about it, we got into like a little deep discussion about it. And he just, like I said, cleared out my mind. And he said, ‘Are you ready?’ And I was like, ‘Yes.’ … It was like, I feel like [if] I would’ve said yes sooner, I probably would’ve gotten it sooner. Yeah. I think he just had it on standby waiting. But no, it didn’t, it wasn’t hard for me to get it at all.” (Participant 16).

Similarly, a participant illustrated the positive influence of their relationship with their healthcare provider on their choice to get vaccinated when they shared:

“You know, but I went back, I went to my doctor… And he and I were talking about it, he was like, ‘You know, this would be a good thing to go ahead and get it while you’re here and go ahead and get it done.’ And he convinced me. He made me feel a little more ease by going ahead and getting it. So, you know, he and I have a good relationship. So, I went ahead and got that shot. [I] actually got to a shingles shot that day, too.” (Participant 13).

Additionally, a participant described the influence of their healthcare provider disclosing their vaccination status as facilitating their uptake, despite their fear of side effects from discussions with family and friends who acted as negative vaccine messengers.

“Because what it was, some people got it and they talk about how sick they got when they took it… So, as I said, I talked with my healthcare providers. And then when my other healthcare provider, my infectious disease doctor said that, she said, when her and her daughter had got it, then, you know, that made me, you know, more comfortable. And that’s when I decided, that right then and there. ‘Okay, give it to me right now, before I change my mind.’” (Participant 9).

#### Barrier: Healthcare Providers as Negative Vaccine Messengers

While some participants shared experiences of healthcare providers acting as positive vaccine messengers, others shared experiences of healthcare providers acting as negative vaccine messengers. One participant described their provider delaying their vaccination, due to a comorbidity, when they described, *“My cardiologist didn’t want me to be vaccinated… He knew if I got COVID it would really take a toll on my health even worse. So*,* when my cardiologist finally cleared me to get vaccinated*,* I went and got vaccinated.”* (Participant 5). Another participant shared their experience with a provider discouraging vaccination when they explained, *“I had a provider say to me*,* ‘You need to stay inside*,* or you need to just not hang out or not go anywhere. Don’t get that vaccine because that vaccine is killing people.'”* (Participant 7). The same participant, who proceeded to get vaccinated, further explained that their primary care physician dissuaded them from getting a booster when they shared, *“I’ve had one*,* but they’re actually recommending another booster because they’re getting more and more strains and the medicine. I went to my primary doctor and she was saying that none of the vaccines*,* [the vaccines] are not fighting off these newer strands.”* (Participant 7).

Amongst the present sample, all of whom received the initial vaccination series, some participants described the undue pressure to vaccinate from their infectious disease doctors, which deterred booster uptake. One participant described how the emphasis on COVID-19 vaccination uptake distracted from their HIV care when they noted, *“Yeah. I guess the focus was so mainly trying to get all the*,* you know*,* positive clients in*,* vaccinated*,* they wasn’t really concentrating on nothing else.”* (Participant 4). Another participant described how the pressure they were put under to vaccinate coerced them into the initial vaccination series and deterred them from further vaccination when they shared:

“I think I feel I was pressured into getting it. Because at first, I was against it…. And then I went for a regular checkup… And then the doctor came in and she’s like, ‘You need the vaccine.’ I was like, ‘No, I don’t want it.’ And she had a nurse come in, did the same thing. And then all of them kind of like crowded me. ‘If you want to live and you want to live a better life with, with your positive status, you need to get these, this vaccine.’ So, it felt pressure[d]. And I still could have said no, but because of the, what I thought was facts, then they were giving me, I felt like I had to… to this day I even haven’t had [a] booster. I haven’t had any issues either. But I’m afraid to put any more of it into my body… So, it’s kind of one of those things that I regret it. But now there’s no taking it back.” (Participant 17).

### Structural Level

#### Facilitator: Vaccination Accessibility

Participants described the role of local pharmacies and organizations in distributing the COVID-19 vaccination, at no cost, as facilitating vaccination uptake. Some participants described receiving the vaccine as part of their HIV care or through a local AIDS Service Organization, such as when they shared, *“[A local ASO] was having a clinic. They actually had this thing to where they offered the clinic for all of their clients and for the general public.”* (Participant 2). As well as, *“Oh*,* they was accessible because my doctor*,* one of my doctors*,* gave it to me.”* (Participant 12). Participants also highlighted the role of their immunocompromised status, due to their HIV status, as facilitating the accessibility of the vaccine. For instance, one participant shared, *“I didn’t really face any*,* any obstacles with it at any point*,* even when I switched and started going to a different pharmacy or whatever. As long as I threw in the healthcare and HIV positive*,* I don’t feel like there was a problem. I’m sure there was for other people. Okay. And I guess I can say*,* I have finally used HIV to my advantage.”* (Participant 19). Similarly, another participant explained, *“I didn’t have an issue as far as*,* I didn’t think I had one as far as getting the vaccine once they opened it up for persons with compromised immune systems. Then it was recommended that I get vaccinated. It’s*,* you know*,* I get the flu vaccine. It’s recommended that I get the pneumonia vaccine. So*,* I get all of those vaccines because of the compromised immune system.”* (Participant 7). One participant described receiving the vaccine through the Department of Veterans Affairs, *“It was relatively accessible because I went to the VA.”* (Participant 3). Other participants shared that they received their vaccination through a pharmacy, for example, *“Oh*,* it was easy. I just went to CVS.”* (Participant 6).

#### Barrier: Vaccination Inaccessibility

A few participants shared vaccination inaccessibility as a barrier to vaccination uptake. This was exemplified when one participant said, *“I did not have transportation to get it. Yeah*,* I didn’t have transportation to get the vaccine.”* (Participant 9). Despite accessibility, wait times acted as an additional barrier to vaccination uptake where one participant described wait times of up to two hours at a local, community vaccination clinic, *“Oh*,* yeah*,* it was easy. I mean*,* I had to*,* I think everyone in the in town of [town name] had to wait like two hours to get their shots because you know*,* the whole city*,* I mean*,* the whole town came to get it*,* or the majority of them did.”* (Participant 23).

#### Facilitator: Vaccination Mandates and Requirements

Many participants described the facilitating role of vaccination mandates and requirements in their uptake of the vaccine. Participants shared that mandates requiring vaccination in the workplace facilitated vaccination, such as when a participant explained, *“…and most of us at work*,* they made it mandatory for us to get it too.”* (Participant 1). As well as when another participant shared, *“So*,* they were pushing it to us because we’re technically always out in the public*,* they considered me*,* at that time*,* a first responder. I don’t know why. So that made me go forward with it.”* (Participant 8). A few participants also shared that the vaccination requirement to travel motivated them to get vaccinated. For instance, one participant described, “*We had to get vaccinated to go on a cruise. So*,* I was going to go on my cruise.”* (Participant 11). One participant highlighted their history of adhering to vaccination requirements to attend school as facilitating their vaccination uptake when they described, *“… look I grew up getting vaccinated. That*,* you had to do that to go to school right? And so*,* we were vaccinated. We got our boosters. We go do it together*,* my mom and I do ours together.”* (Participant 14).

#### Barrier: Misinformation and Misunderstanding

Participants described misinformation and misunderstanding as acting as a barrier to their vaccination uptake, due to its role in their vaccination hesitancy. Common misunderstandings described by participants, that they or members of their networks held, were related to the evolving nomenclature of the virus, speed of vaccination development, potential side effects of the vaccine, as well as the protections afforded from the vaccine, among others. One participant described feeling overwhelmed and hesitant about vaccination because of the various names used to refer to COVID-19. This was demonstrated when they said:

“Because they changed the name of the disease. [The first] version was COVID-19, right? Then its, ah, SARS. What the hell is that? That’s what got me. Yeah, yeah it changed. Yeah, that’s the cousin, sister. Oh, you guys got a big family, huh? Oh, yeah. But yeah, that’s what got me hesitant and that’s the part that got me hesitant. It was just the fact that okay, now you… COVID-19. Now, you got SARS, [Coronavirus] and all this. Yeah, yeah, this is too, too many names. It got to be too much. Overwhelming.” (Participant 15**)**.

Another participant shared the impact of rapid vaccine development on their hesitancy and skepticism in the vaccine when they shared,

“I think the biggest issue was, it seemed like the process to create the vaccine was rushed. I get that they want to protect the American people at all costs, or the world in general, but to rush to make something. Now, that makes me skeptical, because if you can make a vaccine that fast for COVID, why isn’t there a vaccine for HIV? Why isn’t there a vaccine for cancer? Why isn’t there a medicine for cancer? And they seemed like they rushed it along, I guess, to protect the majority of us, but there are more people dying from cancer and HIV. But they’re not putting the concentration on helping, helping those people. So, it seemed like, it seemed like a lot of hidden things going on. And then you hear about how the vaccine was possibly manipulating people’s DNA or changing their blood. So, all that fear in me kind of kept me from wanting to get it.” (Participant 17).

In addition to hesitancy due to the speed of vaccination development, another participant shared their hesitation to vaccinate because of the number of vaccines to choose from: *“Because it was three shots to choose from. So*, *I was like*,* yeah*, *I was very hesitant.”* (Participant 6).

A few participants demonstrated a misunderstanding of the vaccine’s mechanisms to bolster the immune response to infection, rather than to prevent infection altogether. For instance, one participant described their corrected misunderstanding when they said, *“It made me think that if we got the vaccine that we can’t be reinfected*,* yes we can be reinfected.”* (Participant 9). Another participant shared, *“That was baffling to me*,* because I’m like*,* you know*,* what’s the*,* what’s the need to take*,* to be vaccinated if I’m going to get COVID anyway?*” (Participant 7). Further, a participant questioned the continued recommendations to receive vaccination boosters when they explained:

“I will admit that I kept wondering how many boosters we’re gonna have to get. You know, you got the two shots, and I got first two doses. Okay, cool. Then somebody said something about a third one. I’m like, okay, like, so I got the third one. Like, well, we need a fourth one. Okay, well, so what’s going on? Why do we keep needing the shots?” (Participant 13).

Participants also described the pervasive presence of misinformation and mistrust in contributing to their hesitancy. For instance, one participant described vaccination conversations around them as revolving around, *“Weird stuff. It’s a track[er] from the government. They’re tryin’ to put microchips in us. The booster will kill you faster than the virus. You know*,* the virus was basically just made for the government to do just weird stuff. Everybody had their own little*,* own little story about it.”* (Participant 16). Another participant described the influence of vaccine skepticism from their family on their initial hesitancy and then stigmatization, when they shared:

“Yeah, conspiracy theory, conservative family, all that stuff. I’m gonna die of genetic mutation and government implantation and all that stuff… And then after that, everybody just rag[ged] my ass so bad about getting it that I waited a long time to get the second one. And then I ended up getting a booster. But I haven’t gotten any more since because I haven’t gotten sick from it. So, I don’t know.” (Participant 22).

Another participant described the motivation to protect their health as outweighing their hesitancy for the initial vaccination series but confirmed their persistent hesitancy towards the boosters.

“Yeah, I was hesitant about that because, you know, I’m like, damn. Okay, now, yeah, we was gonna start making me think this shit was made in the laboratory but like, what’s really going on? Because I know there’s a lot of misinformation out in the street, but because I am proactive when it comes to my health, I said, ‘Oh let me just go ahead and take it.’ So yeah, so there was a little hesitan[cy] for the booster shots.” (Participant 15).

## Discussion

PWH identified a variety of barriers and facilitators of their COVID-19 vaccination and booster uptake, across all levels of the socioecological model. Primary barriers to vaccination uptake among PWH included misinformation and misunderstanding as well as caution due to the pace and process of the COVID-19 vaccine development [29, 30]. Common facilitators of vaccination were identified as the motivation to protect oneself and others as well as the need to comply with vaccine requirements and mandates. A unique vaccination facilitator among PWH is their acceptance and prioritization of COVID-19 vaccination due to their HIV status. Within the international literature, positive vaccination attitudes, confidence in the vaccine, belief in perceived benefits, perceived behavioral control, perceived high susceptibility, fear of COVID-19 interaction with HIV, and interpersonal support and uptake have been identified as associated with a willingness to vaccinate among PWH [13, 29, 31, 32]. On the other hand, an international systematic review and meta-analysis found that COVID-19 medical mistrust, negative vaccination attitudes, vaccination concerns (i.e., efficacy, safety, and side effects), and distrust of COVID-19 information were related to decreased vaccine acceptance among PWH [33]. Further, within the present study, contrasting findings were found, based on personal experiences with vaccination; messages from family, friends, or healthcare providers; and vaccination accessibility, as to whether they acted as a barrier or facilitator to vaccination. The present study demonstrates unique findings of barriers and facilitators among PWH that may inform future public health communications and interventions.

Situated within the socioecological model, at the individual level, PWH’s perceptions and attitudes towards the COVID-19 vaccine were affected by their HIV status and categorization as an immunocompromised population, which has led to a higher perceived severity of potential consequences resulting from a COVID-19 infection [34]. Further, a novel contribution of this work to the literature is the role of familiarity with injectable antiretroviral therapy (ART), to achieve and maintain an undetectable status, as a vaccination facilitator. The reliance on and success of treatment with ART injectables, as part of HIV care, may positively contribute to vaccine trust among PWH. Alternatively, negative personal experiences with vaccinations and side effects may dissuade PWH from receiving further vaccinations (e.g., COVID-19 boosters).

In addition to the individual level cognitive factors and lived experiences impacting vaccine uptake, discussions and norms within social networks impact PWH’s vaccination decision-making and practices. The favorable vaccination opinions and uptake of trusted family and friends were here found to facilitate PWH’s vaccination uptake. Further, a unique contribution of these findings is the encouragement from family and friends, regardless of their personal vaccination status, for their loved one living with HIV to get vaccinated due to fear of HIV-related complications from a COVID-19 infection. On the other hand, vaccination stigma was also perpetuated within PWH’s social networks and while not dissuading PWH from vaccination, led to the concealment of their vaccination status around negative vaccine messengers. Negative experiences with the initial vaccination series, side effects, and vaccination stigma may have more so acted as a barrier to COVID-19 booster uptake.

Resembling the role of friends and family as vaccination messengers, healthcare providers, at the structural level, serve as trusted messengers who both facilitate and deter COVID-19 vaccination. Due to the involvement in care necessary to maintain an undetectable status, PWH and their healthcare providers have a unique relationship that has the potential to significantly influence patients’ health outcomes [35]. The extant literature has illustrated the correlation between healthcare providers’ perceptions, knowledge, and uptake of vaccination with patient uptake (i.e., if the HCP acts as a positive vaccination messenger the patient is more likely to be vaccinated, and vice versa) [36, 37]. Therefore, there is an urgent need to address vaccination barriers enacted by healthcare providers who act as negative vaccine messengers, particularly in efforts to mitigate and avoid exacerbating health disparities among PWH. As vaccination is understood as a part of HIV care, a framework for the communication and recommendation of vaccines for healthcare providers serving PWH, similar to those guiding healthcare providers’ recommendation of vaccination to parents (e.g., HPV vaccine), is necessitated [38, 39]. A framework of vaccination communication and recommendation is of the utmost importance for healthcare providers serving PWH who experience multiple vulnerabilities (e.g., aging, racial or ethnic minorities, rural residents).

At the furthest level of the socioecological model, the structural level, vaccination mandates and the perpetuation of misinformation influence vaccination uptake. Although government-enacted vaccination mandates (e.g., school-entry vaccination mandates) have been found to increase vaccination coverage, they are likely to be resisted and politicized [40]. Vaccination mandates enacted by smaller, public and private, entities (e.g., employers, travel agencies) may increase vaccination coverage without being met with the same resistance that a state or federal government mandate may be met with. Additionally, misinformation and misunderstanding, as related to low health literacy, act as barriers to positive perceptions of and compliance with vaccination mandates and recommendations [7]. Overall, the politicization of public health recommendations, mandates, COVID-19 vaccinations, and the spread of misinformation have been found to contribute to vaccination hesitancy and deter vaccine and booster uptake [29, 41]. As the pandemic progresses, and COVID-19 boosters continue to be recommended in response to emerging variants and spikes in infection, the role of misinformation and misunderstanding, alongside other factors (e.g., demographics, geography, perceived susceptibility, perceived efficacy, initial vaccination uptake), must be measured and addressed to mitigate their negative effects on booster uptake. The higher uptake of the initial COVID-19 vaccination series as compared to the hesitancy to receive booster vaccinations may be related to the perceived susceptibility, fear, and pressure to vaccinate that characterized the early stages of the pandemic, alongside vaccination recommendations and mandates, but not the later stages.

### Public Health Implications, Strengths and Limitations, and Future Directions

The findings of the present study offer a few, key public health implications and subsequent recommendations. As reflected by the findings from our sample of PWH, in which a majority identified as Black, vaccine hesitancy reflects the effects of systemic racism and medical mistrust, as a result of historic maltreatment, that the literature has identified as related to concerns of vaccine safety, effectiveness, and fears that vaccine recipients were being experimented upon [29, 42]. Due to the level of interaction between PWH and their providers, healthcare providers are uniquely situated to address and alleviate the fears and mistrust of minority PWH. Healthcare providers serving PWH have a responsibility to earn and maintain trust with their patients, which may then positively impact health outcomes, trust in vaccination, and health literacy [43]. Healthcare providers are uniquely situated to address vaccination uptake as they have been identified as trusted messengers amongst patients, including patients who conscribe to vaccination conspiracy beliefs [8, 19]. Further public health efforts are required to improve health communication and health literacy to combat misinformation, misunderstanding, and vaccination stigma through the use of accessible language and transparent messaging. Tailored public health interventions and efforts are necessitated to address the widening COVID-19 and vaccination disparities affecting Black PWH [44, 45].

The present study is characterized by a few strengths and limitations. The study is strengthened by its representation of racial minority populations and PWH living in a Deep South Ending the HIV Epidemic (EHE) priority state. The findings of this work provide a significant contribution to the evolving literature, addressing vaccination hesitancy and booster uptake among a population of multiple vulnerabilities (e.g., age, race/ ethnicity, geography). One methodological strength of the present study is that confirmability and dependability were increased through the use of an audit trail, the engagement of multiple researchers within the data analysis process, and the use of a checklist to report our findings. Additionally, the study’s credibility was enhanced with exemplary quotes and the use of an analysis matrix, as well as the engagement of multiple researchers and experts to confirm their accuracy. The study is also characterized by a few limitations. The recruitment strategy (i.e., purposive, snowball sampling) employed within the present study could limit its transferability to populations that are not a majority Black, older, or situated within a Deep South EHE priority state. The transferability of these findings may be limited because all participants included in the study received the initial vaccination series, were engaged in HIV care, and were on antiretroviral therapy. Additionally, as the sample represented a high average number of years spent living with HIV, the findings may not be transferable to recently diagnosed PWH. Future research is needed to assess the barriers and facilitators to vaccination among PWH not actively engaged in care or who have not yet achieved an undetectable status. A vaccination communication and recommendation framework for PWH should be developed and its implementation evaluated within a variety of populations.

## Conclusions

The present study details a variety of unique barriers and facilitators to vaccination and booster uptake among PWH in the Deep South. First, a familiarity, and success, with injectable antiretroviral therapy has been found to facilitate vaccination through trust, suggesting its role in promoting the uptake of other vaccinations (e.g., COVID-19, monkeypox). Secondly, due to the nature of HIV care and management, there is a unique relationship between healthcare providers and PWH where healthcare providers have the potential to act as trusted, positive vaccine messengers to facilitate uptake and mitigate health disparities exacerbated by COVID-19. Relatedly, at a policy level, there exists the potential for a vaccination communication and recommendation framework to be tailored to healthcare providers who serve PWH (e.g., positive messaging for COVID-19 vaccinations based on a history of injectable antiretroviral therapy). Thirdly, PWH experienced a unique phenomenon where some members of their social networks were reluctant to vaccinate or stigmatized vaccination but simultaneously encouraged PWH to uptake the vaccine due to their HIV status and potential complications from a COVID-19 infection. Finally, despite vaccination hesitancy reported towards the initial vaccination series, amongst a sample who all reported receiving the initial series, the effects of hesitancy were more pronounced in deterring COVID-19 booster uptake. Based on the findings of the present study, PWH face unique barriers and facilitators to COVID-19 vaccination and booster uptake that should be considered when designing and tailoring public health efforts and interventions.

## Appendix

**Analysis Matrix of Emergent Themes**.

**Table:** 

Subcategories	Categories	Subthemes	Theme	Research Question
Fear of complications or death	(+) Need to protect oneself and others	Individual Level	Vaccination is reliant on perceptions of key messengers and availability of resources	What are the hesitancies and perceived barriers and facilitators of COVID-19 vaccination among PWH in South Carolina?
Responsibility to limit spread				
Fear of side effects				
Role of vaccine as a tool to protect HIV from COVID	(+) Vaccination prioritization due to HIV status			
Impact of immunocompromised status				
Fear of COVID triggering HIV				
Receiving Flu Vaccine	(+) Positive personal history of vaccination uptake			
Receiving HIV injectable medications				
Receiving Shingles Vaccine				
Receiving Monkeypox Vaccine				
Experienced negative side effects after first dose	(-) Negative personal history of vaccination experiences			
Experienced negative side effects after second dose				
Encouragement from others	(+) Positive messengers: Family and friends	Interpersonal Level		
Positive experiences of others				
Others experienced negative side effects	(-) Negative messengers: Family and friends			
Opinions of others				
Vaccine stigma				
Established patient-provider history	(+) Positive messengers: Healthcare providers	Institutional Level		
Provider recommendation				
Peer navigation				
Provider vaccination status				
Health planning				
Provider discouragement	(-) Negative messengers: Healthcare providers			
Provider pressure to vaccinate				
Vaccine as a distraction from HIV care				
Provided by HIV infectious disease doctors	(+) Vaccination accessibility	Structural Level		
Provided by local vaccination clinics				
Provided by pharmacies				
Provided by the health department				
Provided by the Department of Veteran Affairs				
Lack of transportation	(-) Vaccination inaccessibility			
Wait times				
Employer mandates	(+) Vaccine requirements and mandates			
Travel requirements				
School mandates				
News	(-) Misinformation and misunderstanding			
Online media				
Skepticism				
Number of vaccine options	(-) Pace and process of vaccine development causing caution			
Speed of development				
Novelty of vaccine				
Associated risks				
Dosage				
Vaccination characteristics				
Perceived risk of vaccination				

Note: (+) indicates a vaccination facilitator and (-) indicates a vaccination barrier
